# Enhanced Interpolated Dynamic DFT Synchrophasor Estimator Considering Second Harmonic Interferences †

**DOI:** 10.3390/s18092748

**Published:** 2018-08-21

**Authors:** Lei Chen, Wei Zhao, Fuping Wang, Qing Wang, Songling Huang

**Affiliations:** 1 Department of Electrical Engineering, Tsinghua University, Beijing 100084, China; l-chen15@mails.tsinghua.edu.cn (L.C.); wangfuping97@mails.tsinghua.edu.cn (F.W.); huangsling@tsinghua.edu.cn (S.H.); 2 Department of Engineering, Durham University, DH1 3LE Durham, UK; qing.wang@durham.ac.uk

**Keywords:** discrete Fourier transform (DFT), digital filter, interpolated dynamic DFT, synchrophasor estimation, second harmonic interference

## Abstract

In the future, phasor measurement units are expected to be applied in distribution networks (DNs) for their control and monitoring. Because of the widely used power electronic devices in DNs, harmonics are widely present in a voltage/current signal. Particularly, second harmonics have the most significant uncertainty contributions to synchrophasor estimation, which is especially true when a short cycle observation window is used for a fast response. Based on the interpolated dynamic discrete Fourier transform (IpD${}^{2}$FT), this paper introduces an enhanced IpD${}^{2}$FT (e-IpD${}^{2}$FT) synchrophasor estimator that considers second harmonic interferences. First, the adaptive equivalent filters of the IpD${}^{2}$FT are given. Based on these, the optimal frequencies where the IpD${}^{2}$FT has the least second harmonic interferences are then searched using an enumeration method, and the e-IpD${}^{2}$FT synchrophasor estimator is accordingly proposed. Instantaneous frequency responses and several simulation tests show that the e-IpD${}^{2}$FT performs much better than the IpD${}^{2}$FT in second harmonic suppression, and can meet the P-class response time requirements and most of the M-class accuracy requirements of the IEEE standard C37.118.1 only over a three-cycle window.

## 1. Introduction

Nowadays, the power flows in distribution networks (DNs) become bidirectional due to the increasing penetrations of distributed energy resources (DERs) (e.g., photovoltaic power generation systems and energy storage systems) in power systems. It is necessary to accurately measure voltage/current phasors, frequencies and even rate of change frequencies (ROCOFs) for DN monitoring and control. To this end, phasor measurement units (PMUs) widely used in transmission networks (TNs) are also expected to be applied in DNs.

According to the IEEE standard C37.118.1-2011 and its amendment standard C37.118.1a-2014 (collectively called the Standard in the following) [1,2], PMUs are divided into two classes for TNs: (1) P-class for fast response applications (i.e., protection); and (2) M-class for high accuracy applications (i.e., measurement and monitoring). In DNs, a PMU is expected to have the above two abilities simultaneously. On the one hand, voltage dips and swells occur frequently in DNs, which requires that PMUs should estimate synchrophasors only over a few cycles. In addition, PMU-based fast fault diagnose and location applications in DNs need fast synchrophasor estimates. On the other hand, large harmonic distortions and frequency deviations can be present in DNs [3,4,5], which requires that PMUs should be robust to large disturbances. However, it is a big challenge to meet these two requirements simultaneously. Harmonic interferences are non negligible in this complex scenario, which is especially true when large frequency deviations are also present in a voltage/current signal [3,4]. Particularly, second harmonics, which can be very high in DNs due to the high penetration of DERs, have the most significant uncertainty contributions [6].

Traditionally, many methods have been proposed to estimate synchrophasors with the consideration of frequency deviations and oscillations [3,7,8,9,10,11,12,13,14]. For example, as for frequency deviations, a very useful tool is the well-known interpolated discrete Fourier transform (IpDFT) [7] and its enhanced version [8]. Regarding oscillations, the Taylor series expansion is widely used in the literature to describe oscillating signals, such as the least square [9], the weighted least square (WLS) [10], the Taylor-DFT [11,12,13], the Taylor${}^{K}$-Kalman filter [14] and the interpolated dynamic DFT (IpD${}^{2}$FT) [3]. However, few of these papers have considered second harmonic interferences. Typically, the IpD${}^{2}$FT is one of the most accurate synchrophasor estimators under oscillation and large frequency deviation conditions [3]. It can obtain synchrophasor, frequency and ROCOF based on the phasor derivative estimates. However, because the second harmonic is the harmonic closest to the window spectrum main lobe, the attenuation around the second harmonic frequency is the lowest. Thus, the IpD${}^{2}$FT suffers from second harmonic interferences, which is especially true when large frequency deviations are also present.

The widely used DFT can suppress second harmonic interferences significantly under synchronous sampling conditions [15]. However, under nonsynchronous conditions, large errors will arise because of the effects of spectral leakage and second harmonic interferences [16]. Recently, a series of methods was proposed to estimate synchrophasors considering second harmonic interferences, such as the improved WLS method [4], the Taylor–Kalman–Fourier filter [17], the Taylor–Fourier transform (TFT) and its improved version [18], i.e., the adaptive TFT [19,20,21]. For example, synchrophasor estimation filters that have a notch filter effect at the second harmonic frequency are designed by the TFT. However, under large frequency deviations, large errors will arise because the second harmonic cannot be filtered clearly. Although the adaptive TFT has a wide stop band around the second harmonic frequency, it needs high computational cost or large memory to calculate filters online or store filters.

In this paper, an enhanced IpD${}^{2}$FT (e-IpD${}^{2}$FT) synchrophasor estimator is proposed considering second harmonic interferences. It only uses a two- or three-cycle data window, and thus its response time is extremely short. It can sufficiently suppress second harmonic interferences, even when large frequency deviations and oscillations are also present. Although higher order harmonics can also be significant in DNs, these harmonics can be suppressed by adopting a suitable window function, e.g., the Hanning window [4]. Interharmonics can also be significant in DNs. Nevertheless, this paper focuses on second harmonic suppression.

## 2. e-IpD${}^{2}$FT Synchrophasor Estimator

This section introduces the e-IpD${}^{2}$FT synchrophasor estimator. First, the classical IpD${}^{2}$FT is briefly recalled. Then, the adaptive equivalent filters of the IpD${}^{2}$FT are given. Next, the optimal frequencies used in the IpD${}^{2}$FT with the least second harmonic interferences are found based on these filters, and the e-IpD${}^{2}$FT is accordingly proposed. Finally, the implementation steps of the e-IpD${}^{2}$FT are summarized.

### 2.1. Signal Model

Generally, a dynamic signal can be expressed as

(1) x(t)=2Re{X1(t)ej(2πft+θ1(t))}

where Re{·} represents the operation selecting the real part of its argument; *f* is the actual fundamental frequency; and $X_{1}\left(t\right)$ and $\theta_{1}\left(t\right)$ are the fundamental amplitude and phase oscillations, respectively. Please note that *f* may differ from the nominal fundamental frequency $f_{0}$ with a static frequency deviation Δf, i.e., $\Delta f=f-f_{0}$. According to [1], a synchrophasor s(t) is defined as a phasor referred to the nominal fundamental frequency, which is given by

(2) $$\begin{array}{cc} s(t) & =\frac{X_{1}\left(t\right)}{\sqrt{2}}e^{j(\theta_{1}\left(t\right)+2\pi \Delta ft)}\\ & =p\left(t\right)e^{j2\pi \Delta ft} \end{array}$$

where p(t) is the so called raw synchrophasor.

### 2.2. Classical IpD${}^{2}$FT

Assume Equation (1) is sampled at sampling rate $f_{s}=f_{0}N_{0}$, and $N_{w}$ samples are obtained in an observation window centered at time $t_{0}=0$, where $N_{0}$ is the sample number of a nominal cycle. Thus, the integer nominal cycle number of the observation window is $c=\lfloor N_{w}/N_{0}\rfloor$. Please note that, to make $t_{0}$ lie in the center of the observation window, $N_{w}$ should be an odd number. The Taylor series expansion is used to approximately express p(t) with a *K*th-order truncation [9]. Then, the signal around time $t_{0}$ can be approximately expressed as

(3) $$\begin{array}{cc} x^{K}\left[n\right]=\frac{\sqrt{2}}{2} & \sum_{k=0}^{K}\frac{1}{k!}{\left(\frac{n}{f_{s}}\right)}^{k}\left[p_{k}e^{j2\pi fn/f_{s}}+p_{k}^{*}e^{-j2\pi fn/f_{s}}\right]\\ & n=-\left(N_{w}-1\right)/2,\ldots ,0,\ldots ,\left(N_{w}-1\right)/2 \end{array}$$

where the superscript ${}^{*}$ represents the conjugate operation; and $p_{k}$ is the *k*th-order derivative of p(t) calculated at time $t_{0}$. Computing the windowed discrete-time Fourier transform (DTFT) of Equation (3), we have

(4) $$\begin{array}{cc} X^{K}\left(f_{b}\right) & =\frac{\sqrt{2}}{N_{w}}\sum_{n=-(N_{w}-1)/2}^{(N_{w}-1)/2}x^{K}\left[n\right]w\left[n\right]e^{-j2\pi f_{b}n/f_{s}}\\ & =\sum_{k=0}^{K}\left[p_{k}W_{k}\left(f_{b}-f\right)+p_{k}^{*}W_{k}\left(f_{b}+f\right)\right]\;b=1,2,3 \end{array}$$

where $f_{b}$ (with b=1,2,3) is a bin frequency (with $f_{1}=\frac{\left(c-1\right)f_{s}}{N_{w}},f_{2}=\frac{cf_{s}}{N_{w}}$ and $f_{3}=\frac{\left(c+1\right)f_{s}}{N_{w}}$), w[·] is a window function, and

(5) $$\begin{array}{c} W_{k}\left(f_{b}\right)=\frac{1}{N_{w}}\sum_{n=-(N_{w}-1)/2}^{(N_{w}-1)/2}\frac{1}{k!}{\left(\frac{n}{f_{s}}\right)}^{k}w\left[n\right]e^{-j2\pi f_{b}n/f_{s}} \end{array}$$

is a function related to the derivative order *k* (with *k* = 0, 1, ..., *K*) and $f_{b}$ (with b=1,2,3). Truncate the Taylor series expansion to the second order (K=2). Then, Equation (4) can be rearranged as

(6) $$\begin{array}{cc} \left[\begin{array}{cc} W{}_{R} & W{}_{I}\\ W{{}_{I}}^{*} & W{{}_{R}}^{*} \end{array}\right]\left[\begin{array}{c} p\\ p^{*} \end{array}\right] & \approx \left[\begin{array}{c} X\\ X^{*} \end{array}\right] \end{array}$$

with $X\in C^{3}$ being a column vector containing the windowed DTFT of x[n] at the three bin frequencies; $p\in C^{3}$ and $p^{*}\in C^{3}$ being two column vectors containing $p_{k}$ and its conjugate phasor $p_{k}^{*}$ (with k=0,1,2), respectively; and $W{}_{R}\in C^{3\times 3}$ and $W{}_{I}\in C^{3\times 3}$ being two matrices containing $W_{k}\left(f_{b}-f\right)$ and $W_{k}\left(f_{b}+f\right)$ computed at different orders *k* (with k=0,1,2) and different frequencies $f_{b}$ (with b=1,2,3). Additionally, Equation (6) can be rearranged as

(7) $$\begin{array}{c} WP\approx Y \end{array}$$

where W is a matrix consisting of $W{}_{R}$, $W{}_{I}$ and their conjugate matrices; P is a column vector consisting of the two vectors p and $p^{*}$; and Y is a column vector consisting of two vectors X and $X^{*}$. Obviously, because the three bin frequencies used in the windowed DTFT are unequal, $W\in C^{6\times 6}$ is a full rank matrix. Then, P can be easily estimated by

(8) $$\begin{array}{c} \hat{P}=W^{-1}Y \end{array}$$

Thus, the raw synchrophasor ${\hat{p}}_{0}$ and its derivatives ${\hat{p}}_{1}$ and ${\hat{p}}_{2}$ are obtained. Then, the fundamental frequency and ROCOF can be estimated by [9]

(9) f^(t0)=f+12π·Im{p^1p^0*}|p^0|2

(10) ROCOF^(t0)=12πIm{p^0*p2}|p^0|2−1πRe{p^0*p^1}Im{p^0*p^1}|p^0|4

where Im{·} represents the operation selecting the imaginary part of its argument. Accordingly, the frequency deviation can be estimated by

(11) $$\begin{array}{c} \Delta \hat{f}\left(t_{0}\right)=\hat{f}\left(t_{0}\right)-f_{0} \end{array}$$

In this way, the estimated synchrophasor $\hat{s}\left(t_{0}\right)$ can be obtained according to Equation (2). It should be noted that the actual fundamental frequency *f* is usually unknown. It is originally assumed as $f_{0}$ and then three iterations are needed to get an accurate estimate [3].

Obviously, the second harmonic interferences are not considered in [3]. If the second harmonic is significant, it will lead to a large interference component in Y, and large differences will be present between WP and Y. As a result, large errors will arise (see Equation (7)). Compared with other harmonics, the second harmonic is closest to the window spectrum main lobe. Thus, it has the most significant uncertainty contribution to synchrophasor estimation.

### 2.3. Adaptive Equivalent Filters of the IpD${}^{2}$FT

As is well known, PMU is a typical discrete-time system, and a PMU using the IpD${}^{2}$FT method can be seen as a linear time-invariant system during each iteration. Thus, during each iteration, the phasor derivative ($p_{0}$, $p_{1}$ and $p_{2}$) estimators can be equivalent to three digital finite-impulse-response (FIR) filters. Then, the ability of second harmonic interference suppression of the IpD${}^{2}$FT can be evaluated based on these adaptive equivalent filters. Consider that, in Equation (8), Y can be expressed in linear convolution, which is given by

(12) $$\begin{array}{c} Y=\left[\begin{array}{c} g_{1}\left[n\right]⊗x\left[n\right]\\ g_{2}\left[n\right]⊗x\left[n\right]\\ g_{3}\left[n\right]⊗x\left[n\right]\\ g_{1}^{*}\left[n\right]⊗x\left[n\right]\\ g_{2}^{*}\left[n\right]⊗x\left[n\right]\\ g_{3}^{*}\left[n\right]⊗x\left[n\right] \end{array}\right] \end{array}$$

where ⊗ denotes the operation of linear convolution, and

(13) $$\begin{array}{c} g_{b}\left[n\right]=\frac{\sqrt{2}}{N_{w}}\cdot w\left[n\right]\cdot e^{j2\pi f_{b}n/f_{s}}\;b=1,2,3 \end{array}$$

is the equivalent filter of the windowed DTFT. Assume the six row vectors of the matrix $W^{-1}$ are $R_{i}$ (with i=0,…,5); $R_{i}\left[q\right]$ represents the *q*-th element of $R_{i}$, which is related to $W_{k}\left(f_{b}-f\right)$ and $W_{k}\left(f_{b}+f\right)$, and can be seen as the gain of the corresponding DTFT value Y[q]; $\hat{P}\left[i+1\right]$ represents the (i+1)-th element of P, i.e., the phasor derivatives and their conjugate phasors. Then, Equation (8) can also be expressed as

(14) $$\begin{array}{c} \hat{P}\left[i+1\right]=\sum_{q=1}^{6}R_{i}\left[q\right]Y\left[q\right]\\ =\sum_{q=1}^{3}R_{i}\left[q\right]\cdot \left(g_{q}\left[n\right]⊗x\left[n\right]\right)+\sum_{q=4}^{6}R_{i}\left[q\right]\cdot \left(g_{q-3}^{*}\left[n\right]⊗x\left[n\right]\right)\\ \;\;\;\;\;\;\;\;\;\;i=0,\ldots ,5 \end{array}$$

In this way,

(15) $$\begin{array}{c} h_{i}\left[n\right]=\sum_{q=1}^{3}R_{i}\left[q\right]\cdot g_{q}\left[n\right]+\sum_{q=4}^{6}R_{i}\left[q\right]\cdot g_{q-3}^{*}\left[n\right]\\ n=-\left(N_{w}-1\right)/2,\ldots ,0,\ldots ,\left(N_{w}-1\right)/2\\ i=0,1,2 \end{array}$$

are the adaptive equivalent FIR filters for $p_{0}$, $p_{1}$ and $p_{2}$ estimation, respectively. According to Equations (4), (13) and (15), these filters are related to the three DTFT frequencies $f_{b}$ (with b=1,2,3) and the fundamental frequency *f*. During each iteration, the IpD${}^{2}$FT modifies the fundamental frequency *f* in its signal model, and W (or $R_{i}\left[q\right]$) is recalculated. Then, the adaptive equivalent filters are redesigned.

Theoretically, if any three different frequencies around *f* are used in the windowed DTFT, W will be a full rank matrix, and Equation (7) can be successively solved. Thus, not only the bin frequencies but also other three frequencies around *f* can be used to design the above adaptive filters. Obviously, when different DTFT frequencies are used, there will be different second harmonic interference components in Y. That is also to say, the adaptive filters (thus the IpD${}^{2}$FT) will have different performances on second harmonic suppression [22]. Thus, we can find optimal DTFT frequencies to design adaptive filters with the strongest second harmonic suppression. Then, the total vector error (TVE), frequency error (FE) and ROCOF error (RFE) will be controlled at a low level.

### 2.4. DTFT Frequency Selection for Accuracy Enhancement of the IpD${}^{2}$FT

This subsection first selects the optimal DTFT frequencies for the no frequency deviation condition, i.e., $f=f_{0}$. Then, the solution to the frequency deviation condition is given.

When $f=f_{0}$, the IpD${}^{2}$FT does not need any iteration. Then, it is true that the IpD${}^{2}$FT can be seen as a bank of FIR filters given in Equation (15) (no longer the adaptive ones). We can use the frequency responses of these equivalent filters to evaluate the TVE, FE and RFE of the IpD${}^{2}$FT. According to [23], if Equation (1) is stationary (i.e., the fundamental magnitude and phase oscillations are ignored), the relationship between the IpD${}^{2}$FT’s TVE (FE or RFE) and its frequency response can be given by (see Appendix A).

(16) $$TVE\leq \frac{\sqrt{2}}{2}\left\{\right|H_{0}\left(f_{0}\right)-\sqrt{2}\left|+\right|H_{0}\left(-f_{0}\right)\left|+r(\right|H_{0}\left(2f_{0}\right)\left|+\right|H_{0}\left(-2f_{0}\right)\left|)\right\}$$

(17) $$FE\leq \frac{\sqrt{2}}{2}\cdot \frac{1}{2\pi }\left(\right|H_{1}\left(f_{0}\right)\left|+\right|H_{1}\left(-f_{0}\right)\left|\right)+\frac{r}{2\pi }\left(\right|H_{1}\left(2f_{0}\right)\left|+\right|H_{1}\left(-2f_{0}\right)\left|\right)$$

(18) $$RFE\leq \frac{\sqrt{2}}{2}\cdot \frac{1}{2\pi }\left(\right|H_{2}\left(f_{0}\right)\left|+\right|H_{2}\left(-f_{0}\right)\left|\right)+\frac{r}{2\pi }\left(\right|H_{2}\left(2f_{0}\right)\left|+\right|H_{2}\left(-2f_{0}\right)\left|\right)$$

where $H_{i}\left(f\right)$(with i=0,1,2) represents the frequency response of $h_{i}\left[n\right]$ (with i=0,1,2) at frequency *f*; and *r* is the magnitude ratio of the second harmonic to the fundamental. After some algebra and simplifications (see Appendix B), Equations (16)–(18) can be rewritten as

(19) $$\begin{array}{c} TVE\leq \frac{\sqrt{2}}{2}\cdot r\left|H_{0}\left(2f_{0}\right)\right| \end{array}$$

(20) $$\begin{array}{c} FE\leq \frac{\sqrt{2}}{2}\cdot \frac{r}{2\pi }\left|H_{1}\left(2f_{0}\right)\right| \end{array}$$

(21) $$\begin{array}{cc} RFE & \leq \frac{\sqrt{2}}{2}\cdot \frac{r}{2\pi }\left|H_{2}\left(2f_{0}\right)\right| \end{array}$$

Thus, the IpD${}^{2}$FT’s TVE, FE and RFE upper bounds are mainly determined by the filters’ attenuation at the second harmonic frequency. Based on the enumeration method, three optimal frequencies can be found to minimize the sum of the TVE, FE and RFE upper bounds, i.e.,

(22) $$\begin{array}{c} e=\frac{\sqrt{2}}{2}\left\{r\right|H_{0}\left(2f_{0}\right)|+\frac{r}{2\pi }\left(\right|H_{1}\left(2f_{0}\right)\left|+\right|H_{2}\left(2f_{0}\right)\left|)\right\} \end{array}$$

The implementation details of the enumeration method are as follows. Generally, to make W a full rank matrix, the three frequencies cannot be the same. In this paper, the minimum deviations between each of the two frequencies are set at 1 Hz. Additionally, to reduce the infiltration from the image fundamental tone and second harmonic component [6], the three frequencies need to be close to *f*. In this paper, they are limited in a frequency band of (25, 75) Hz. Finally, the step for optimal frequencies searching is set at 0.2 Hz.

It should be highlighted that the optimal frequencies are for the no frequency deviation condition. However, in practice, the fundamental frequency frequently deviates from the nominal value. To deal with this problem, we can take the following measures:

- Compute W and Y using the optimal frequencies $f_{b}$ of the no frequency deviation condition, and estimate the actual fundamental frequency *f* using Equation (9).
- Modify $f_{b}$ as $f_{b}+2\Delta f$ (with b=1,2,3), and recompute W and Y to estimate the actual fundamental frequency again.
- Repeat the above two steps twice (i.e., another two iterations) for high accuracy.

**Figure 1:**
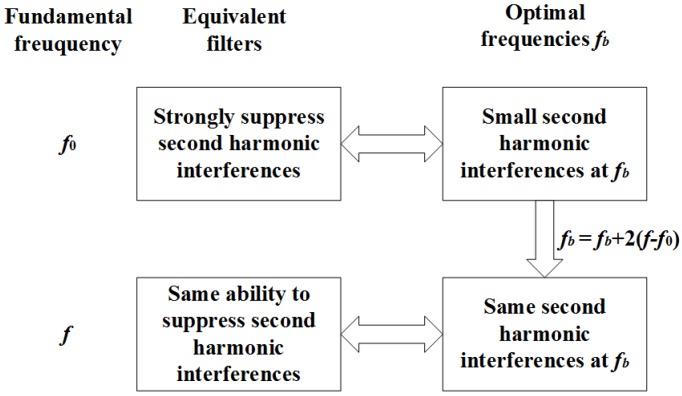
Illustration of the principle.

**Figure 2:**
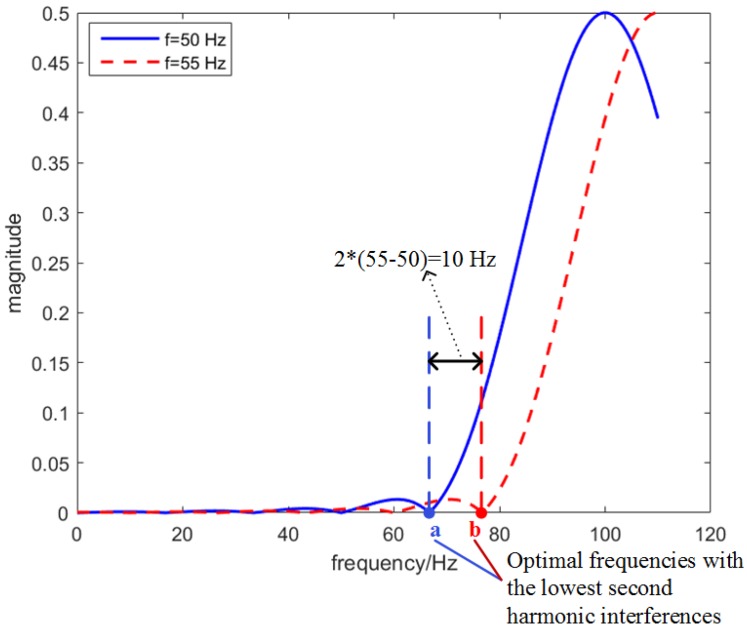
Illustration of the second harmonic interference. The red and blue lines are second harmonic interferences at different frequencies. The image second harmonic component is ignored. Only one bin used in the IpD${}^{2}$FT is illustrated.

In this way, the new adaptive filters will have similar second harmonic suppression abilities as the filters for the no frequency deviation condition. The reason is shown in Figure 1 and Figure 2. When the adaptive equivalent filters can sufficiently suppress second harmonic interferences, it is equivalent to the second harmonic interferences at $f_{b}$ being very small (see the red line in Figure 2) [22]. When the fundamental frequency has a deviation of Δf, the new optimal frequencies should deviate $f_{b}$ with a deviation of 2Δf to keep the ability of second harmonic suppression (see the blue and red bins in Figure 2).

In Table 1, the optimal DTFT frequencies for the no frequency deviation condition are listed. In this paper, short cycle windows, i.e., two- and three-cycle windows, are considered for fast responses. The Hanning and Hamming windows are both adopted for illustration. In Table 1, we can see that the optimal DTFT frequencies for two-cycle windows (both the Hanning and Hamming window) are very small. This is because the DTFT values at small frequencies will have small interferences from second harmonics (see Figure 2).

### 2.5. Implementation Steps of the e-IpD${}^{2}$FT

**Figure 3:**
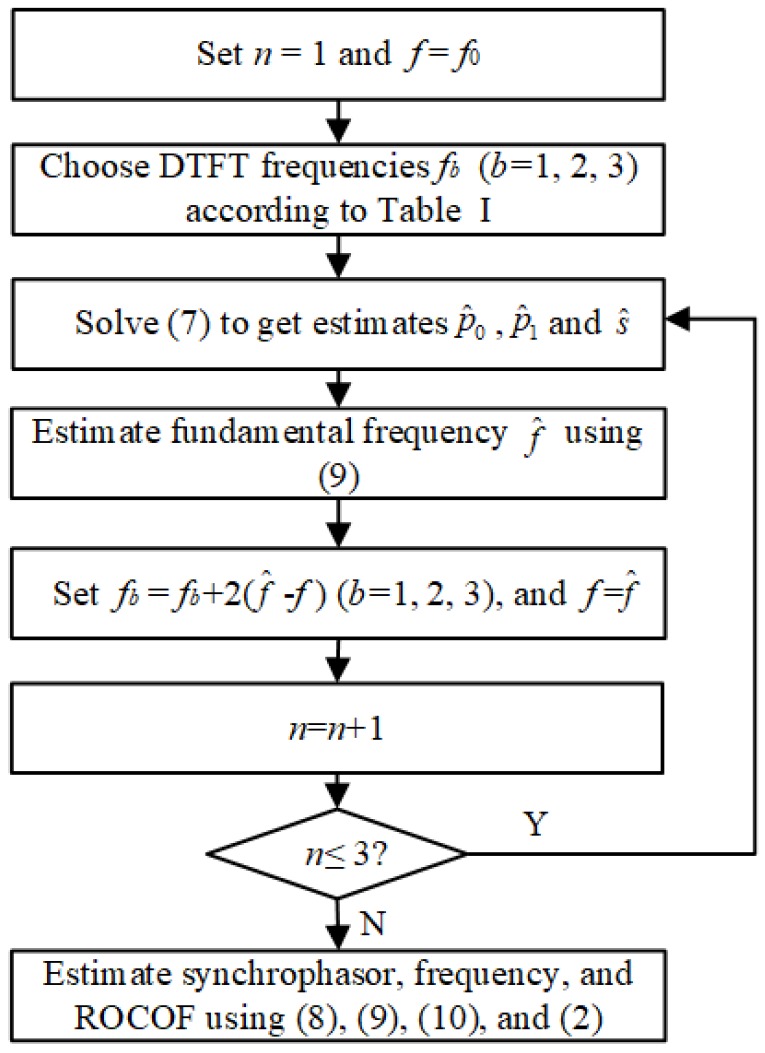
Implementation steps of the e-IpD${}^{2}$FT.

The implementation steps of the e-IpD${}^{2}$FT are summarized in Figure 3. To suppress second harmonic interferences under frequency deviation conditions, a first estimation of fundamental frequency should be carried out, and then the three DTFT frequencies are adaptively modified. In this way, the estimated fundamental frequency is not only used in signal model foundation but also in DTFT frequency modification. For a high accuracy, we take three iterations to modify the fundamental frequency and thus the DTFT frequencies.

Based on the optimal DTFT frequency selection and adaptive modification, the e-IpD${}^{2}$FT can suppress second harmonic interferences. Even when the second harmonic interferences and frequency deviations occur simultaneously, the e-IpD${}^{2}$FT can still estimate the synchrophasor, frequency and ROCOF with a high accuracy.

### 2.6. Computational Complexity

The main computations of the e-IpD${}^{2}$FT and IpD${}^{2}$FT are generating matrices (W, Y) and solving Equation (7). Assume there are $N_{w}$ samples in the observation window; the iteration number is *n*; and the Taylor truncation order is K=2. Then, the main computations of the two methods are listed in Table 2. Compared with the IpD${}^{2}$FT, the e-IpD${}^{2}$FT’s DTFT frequencies are adaptively modified at each iteration. Thus, the DTFT computation will change at each iteration, and the computation time of the e-IpD${}^{2}$FT will be longer than the IpD${}^{2}$FT. However, because only three iterations are carried out, the overall complexity of the e-IpD${}^{2}$FT will still be quite small. If $N_{0}=40$, then the overall floating-point operations of the two- and three-cycle e-IpD${}^{2}$FT are only 45,468 and 94,122, respectively. Thus, even if a low performance digital signal processor is used, the computation time will still be very short.

## 3. Instantaneous Frequency Response

**Figure 4:**
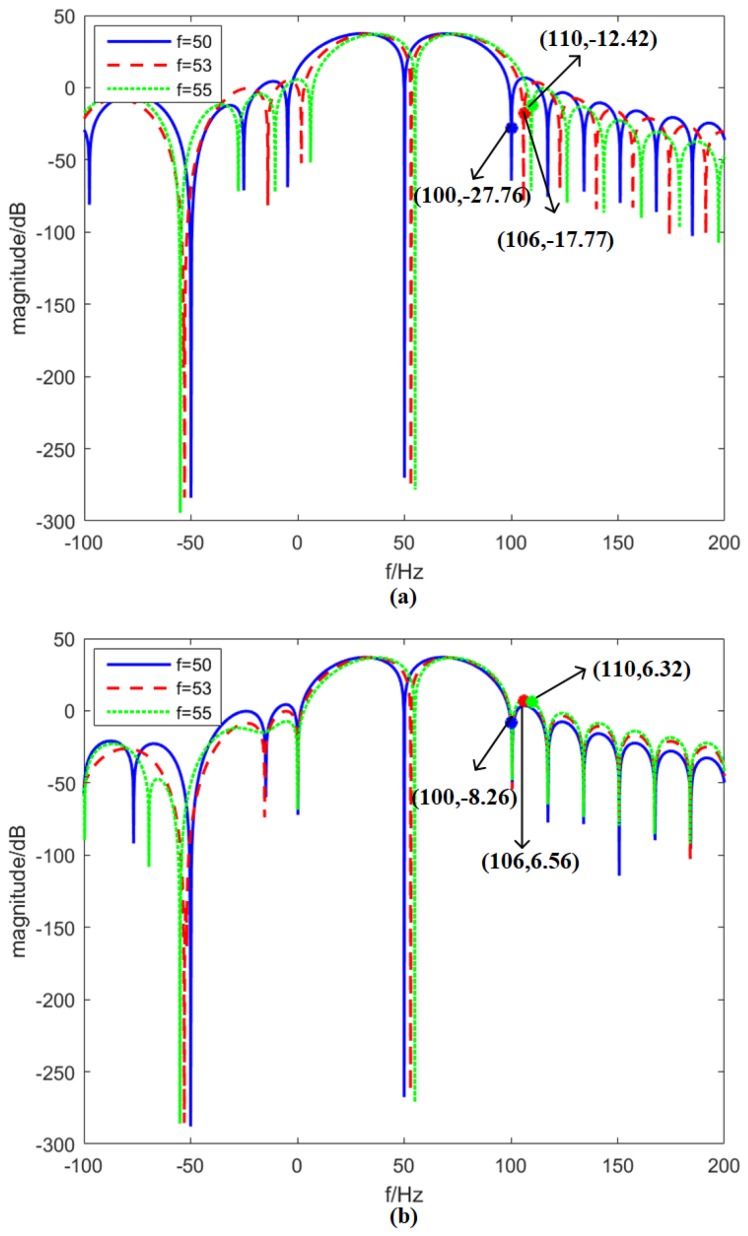
Instantaneous frequency response of the e-IpD${}^{2}$FT and IpD${}^{2}$FT for $p_{1}$ estimation. The three-cycle Hanning window is chosen for illustration. *f* is set at 50, 53 and 55 Hz, respectively: (**a**) e-IpD${}^{2}$FT; and (**b**) IpD${}^{2}$FT.

This section mainly compares the performances of the IpD${}^{2}$FT and e-IpD${}^{2}$FT for second harmonic suppression. In Figure 4, the instantaneous frequency responses of the e-IpD${}^{2}$FT and IpD${}^{2}$FT are shown. As is known, frequency and ROCOF estimates are more sensitive to second harmonic interferences. This section selects the filter on $p_{1}$ estimation for illustration. In Figure 4a, we can see that, when the fundamental frequency is set at 50, 53 and 55 Hz, the second harmonic attenuation of the e-IpD${}^{2}$FT’s adaptive equivalent filter is 27.76, 17.77 and 12.42 dB, respectively. By contrast, the second harmonic attenuation of the IpD${}^{2}$FT’s adaptive equivalent filter is only 8.26, −6.56 and −6.32 dB, respectively (see Figure 4b). The second harmonic attenuation of the e-IpD${}^{2}$FT’s adaptive equivalent filter is always bigger than 10 dB, and is about 20 dB larger than the IpD${}^{2}$FT’s. This evidence shows that the e-IpD${}^{2}$FT can suppress second harmonic interferences, even under large frequency deviation conditions.

It is interesting that Figure 4a,b shows different shapes around second harmonic frequency. The reason is shown in Figure 1 and Figure 2. When different frequencies are used in the IpD${}^{2}$FT, the DTFT values in Y will have different interferences from the second harmonic. If the optimal frequencies are used, the interferences will be very small, and the adaptive equivalent filters can strongly suppress the second harmonic. Moreover, under frequency deviation conditions, the optimal frequencies will be adaptively modified, and the second harmonic will still be sufficiently suppressed.

## 4. Simulation Tests

To evaluate and compare the performances of the e-IpD${}^{2}$FT and IpD${}^{2}$FT, a series of simulation tests are carried out. First, canonical tests stated in the Standard are carried out to show that the e-IpD${}^{2}$FT can meet basic requirements of the Standard. Next, peculiar conditions where multiple disturbances occur simultaneously are considered to show the e-IpD${}^{2}$FT’s robustness to large second harmonic interferences. Finally, a real world example is taken to show the practical values of the e-IpD${}^{2}$FT.

More details about the tests are as follows. (1) Two- and three-cycle windows are particularly considered in this paper. (2) The Hanning and Hamming windows are both adopted for illustration. (3) All test signals are sampled at 2000 Hz (for 50-Hz system). (4) The initial phases of all possible components (e.g., the fundamental and the second harmonic) are randomly selected within [0,2π] rad. (5) At distribution level, the level of harmonics or other disturbances can be very high. Thus, all signal parameters, such as frequency deviation, harmonic distortion level, modulation level, modulation frequency and linear frequency ramp rate, are set at the maximum values of M-class tests stated in the Standard (corresponding to the worst conditions). (6) As for accuracy tests, the M-class TVE, FE and RFE limits in the Standard are referred with reporting rate RR = 50 frames/s (in China, RR is generally set at 50 frames/s). (7) Regarding responsiveness tests, the P-class response time limits in the Standard are referred for transient tests.

### 4.1. Canonical Tests

In this subsection, a set of canonical tests stated in the Standard, including frequency deviation, harmonic distortion, amplitude and phase modulations (AM + PM), frequency ramp and step changes, are carried out to verify the performances of the proposed method. The corresponding results are shown in Table 3. As shown, although sometimes the results of the e-IpD${}^{2}$FT are worse than the IpD${}^{2}$FT, they are always much smaller than the corresponding limits. In this respect, the e-IpD${}^{2}$FT can be used for practical applications. The differences between the results of the e-IpD${}^{2}$FT and IpD${}^{2}$FT are caused by their different instantaneous frequency responses.

In addition to the above tests, out-of-band interferences with frequencies within the interval [10, $f_{0}-RR/2]$ and $\left[f_{0}+RR/2,100\right]$ should also be considered to test the proposed method. These frequencies are close to the optimal frequencies and the bin frequencies, which makes the interferences too high. As a result, neither the e-IpD${}^{2}$FT nor the IpD${}^{2}$FT can meet the Standard’s requirements. The performances of the e-IpD${}^{2}$FT are similar to the IpD${}^{2}$FT as reported in [3]. These interferences can be suppressed by adopting a long observation window [3], but this is not the subject of this paper.

### 4.2. Frequency Deviation + Second Harmonic

In DNs, frequency deviations and second harmonic interferences may occur simultaneously. To test the proposed method’s robustness to this condition, a signal with a −5-Hz frequency deviation (*f* = 45 Hz) and a 10% second harmonic is considered. In the following, the abbreviation “frequency deviation + second harmonic” is used to denote the test defined above. Similar abbreviations used in the following have the similar meanings. This is the worst test condition stated in the Standard.

The maximum TVEs, |FE|s and |RFE|s returned by the e-IpD${}^{2}$FT and IpD${}^{2}$FT are shown in Table 4. In Table 4, we can see that the e-IpD${}^{2}$FT is much more accurate than the IpD${}^{2}$FT. This is because the e-IpD${}^{2}$FT can select optimal frequencies and adaptively modify them under frequency deviations. Thus, even under large frequency deviations, the e-IpD${}^{2}$FT can still sufficiently suppress the second harmonic interferences. Additionally, when a three-cycle window is used, the maximum TVEs, |FE|s and |RFE|s of the e-IpD${}^{2}$FT are much smaller than the related limits of the Standard. In this respect, the e-IpD${}^{2}$FT has a better performance than the IpD${}^{2}$FT, which needs a four-cycle window to achieve the same performance [3].

### 4.3. Frequency Deviation + Second Harmonic + Modulation

In this subsection, another complex scenario that may occur in DNs is considered. Specifically, not only the −5-Hz frequency deviation and 10% second harmonic but also the fundamental amplitude and phase modulations are considered. The modulation level and frequency are set at 0.1 and 5 Hz, respectively. According to the Standard, this is also the worst condition for such a test.

In Table 5, the results returned by the e-IpD${}^{2}$FT and IpD${}^{2}$FT under such condition are shown. Please note that the e-IpD${}^{2}$FT is much more accurate than the IpD${}^{2}$FT because it can significantly suppress second harmonic interferences. In addition, the maximum TVEs, |FE|s and |RFE|s returned by the three-cycle e-IpD${}^{2}$FT are still smaller than the corresponding limits of the Standard.

### 4.4. Other Complex Scenarios

In some complex scenarios, not only the second harmonics but also the third or higher-order harmonics may be present in a voltage/current signal. Generally, only the second and third harmonics have strong impacts on synchrophasor estimation. Higher-order harmonics can be sufficiently suppressed by the Hanning window [4]. Meanwhile, additive wide band noise should also be considered. In this subsection, two typical scenarios are considered: (a) joint effects of −5-Hz frequency deviation, 10% second harmonic and third harmonic; and (b) joint effects of −5-Hz frequency deviation, 10% second harmonic and 60-dB noise.

The TVEs, |FE|s and |RFE| of both methods (maximum in Scenario (a), mean and standard deviation values in Scenario (b)) are reported in Table 6. Compared with Table 4, several conclusions can be drawn. Although the third harmonic or wide band noise are also present, the three-cycle e-IpD${}^{2}$FT can still meet the corresponding requirements of the Standard. However, the IpD${}^{2}$FT cannot meet the corresponding requirements. In the test of “Fre. Dev. + 2nd Harm. + 60 dB Noise”, the e-IpD2FT with respect to the IpD2FT shows a remarkable performance improvement for the RFE. When in the presence of second harmonics and frequency deviation, large interferences will be present at bin frequencies, whereas they are negligible at the adaptive selected frequencies. Because the RFE is the rate of FE, it is more sensitive to such interferences. As a result, the e-IpD${}^{2}$FT has much smaller |RFE|s (mean and standard deviation values) than the IpD${}^{2}$FT.

### 4.5. Sampling Rate

As is known, the optimal frequencies given in Table 1 are obtained in a sampling frequency of 2000 Hz. In this subsection, the performances of the e-IpD${}^{2}$FT under different sampling rates are tested (2000, 2400, 4000 and 4800 Hz). The fundamental frequency is set at 51 Hz (1-Hz frequency deviation), and the magnitude ratio of the second harmonic to the fundamental is set at 5%.

The results are shown in Table 7. As shown, although the sampling rate varies, the maximum TVEs, |FE|s and |RFE|s returned by the e-IpD${}^{2}$FT do not have any variation. By contrast, the results returned by the IpD${}^{2}$FT have a few changes. The reason is as follows. For the e-IpD${}^{2}$FT, the DTFT frequencies are the same in all tests. However, the IpD${}^{2}$FT modifies its DTFT frequencies ($f_{b}=\frac{\left(c-1\right)f_{s}}{N_{w}},\frac{cf_{s}}{N_{w}}$ and $\frac{\left(c+1\right)f_{s}}{N_{w}}$) with the sampling rate variations. Thus, the DTFT values (Y) of the e-IpD${}^{2}$FT are the same in all tests, whereas the DTFT values of the IpD${}^{2}$FT are not the same. Anyway, the e-IpD${}^{2}$FT is robust to sampling rate variations.

### 4.6. A Real World Example

To demonstrate the real benefit of the e-IpD${}^{2}$FT, a real world example in industry is taken in this paper. According to [24], transformer current during inrush can have a second harmonic up to 63% of the fundamental. Meanwhile, higher-order harmonics can also be present. In this subsection, such a complex scenario is considered. Harmonic levels are set according to [24] (see Table 8). The fundamental frequency is set at 49 Hz, and 60 dB wide band noise is added to the signal. Accordingly, the results are reported in Table 9. We can see that, even under such large harmonic disturbance conditions, the mean TVE, |FE| and |RFE| of the e-IpD${}^{2}$FT are only 0.55%, 0.01 Hz and 0.98 Hz/s, respectively, which are smaller than the corresponding limits of the Standard. However, the IpD${}^{2}$FT cannot meet the corresponding requirements. This example shows the significant practical values of the e-IpD${}^{2}$FT.

## 5. Conclusions

This paper proposes a novel synchrophasor estimator, especially for DNs, where the most significant uncertainty contribution is the second harmonic. The adaptive equivalent filters of the IpD${}^{2}$FT are given in this paper, which are used for finding optimal frequencies. The e-IpD${}^{2}$FT has a fast response, and can meet P-class response time requirements because only a few cycles are used. In addition, it has a high accuracy, and can meet most of the M-class accuracy requirements only over a three-cycle observation window, even when oscillation, large frequency deviation and harmonic distortions occur simultaneously in a voltage/current signal. A real world example is taken to show the real benefit of the e-IpD${}^{2}$FT.

## Figures and Tables

**Table 1:** Optimal DTFT frequency sets for different windows and window lengths with $f=f_{0}$. The sampling frequency is set at 2000 Hz (for 50-Hz system).

c	Hanning	Hamming
2	{25.0, 26.0, 27.2}	{28.2, 33.8, 46.0}
3	{29.2, 53.0, 66.2}	{35.2, 45.4, 65.0}

**Table 2:** Computational complexity of the e-IpD${}^{2}$FT and IpD${}^{2}$FT. The computation types + and × denote complex addition and multiplication, respectively.

Method	Comp.	Comp. Type		
		+	×	exp
e-IpD${}^{2}$FT	W	$6n(N_{w}-1)$	$6nN_{w}$	$6nN_{w}$
	Y	$3n(N_{w}-1)$	$3nN_{w}$	$3nN_{w}$
	(7)	130n	412n	–
IpD${}^{2}$FT	W	$6n(N_{w}-1)$	$6nN_{w}$	$6nN_{w}$
	Y	$3(N_{w}-1)$	$3N_{w}$	$3N_{w}$
	(7)	130n	412n	–

**Table 3:** Canonical test results and the corresponding thresholds in the standard. The Hanning window is adopted in the e-IpD${}^{2}$FT and IpD${}^{2}$FT. The observation window is three cycles long. The results of the amplitude step change test are expressed in nominal cycles. In other tests, the TVEs (%), |FE|s (Hz) and |FE|s (Hz/s) are given in maximum values.

**Parameter**	**Fre. Dev.**			**Harm. Dist.**			**AM + FM**		
	**std**	**e-IpD**${}^{2}$**FT**	**IpD**${}^{2}$**FT**	**std**	**e-IpD**${}^{2}$**FT**	**IpD**${}^{2}$**FT**	**std**	**e-IpD**${}^{2}$**FT**	**IpD**${}^{2}$**FT**
TVE	1	0.00	0.00	1.00	0.09	0.05	3.00	0.03	0.01
|FE|	0.005	0.00	0.00	0.03	0.00	0.01	0.30	0.02	0.02
|RFE|	0.1	0.00	0.00	–	0.06	1.73	14.00	0.95	0.92
**Parameter**	**Amp. Step Change (±10%)**			**Ph. Step Change (**$\pm \frac{\pi }{18}$**)**			**Fre. Ramp**		
	**std**	**e-IpD**${}^{2}$**FT**	**IpD**${}^{2}$**FT**	**std**	**e-IpD**${}^{2}$**FT**	**IpD**${}^{2}$**FT**	**std**	**e-IpD**${}^{2}$**FT**	**IpD**${}^{2}$**FT**
TVE	2.00	0.82	0.82	2.00	1.60	0.95	1.00	0.00	0.00
|FE|	4.50	2.35	2.35	4.50	2.38	2.57	0.01	0.00	0.00
|RFE|	6.00	2.70	2.70	6.00	2.78	2.53	0.20	0.00	0.00

**Table 4:** Maximum TVEs (%), |FE|s (Hz) and |RFE|s (Hz/s) returned by the e-IpD${}^{2}$FT and IpD${}^{2}$FT in the “frequency deviation + second harmonic” test. Gray cells refer to the results beyond the boundaries of the M-class requirements of the standard.

Parm.	Std.	c	Hanning		Hamming	
			e-IpD${}^{2}$FT	IpD${}^{2}$FT	e-IpD${}^{2}$FT	IpD${}^{2}$FT
TVE	1	2	1.97	7.47	0.47	7.14
		3	0.10	2.42	0.11	1.61
|FE|	0.025	2	0.36	1.21	0.11	1.15
		3	0.01	0.11	0.01	0.12
|RFE|	–	2	120.66	472.07	14.97	423.75
		3	0.56	88.66	2.97	52.26

**Table 5:** Maximum TVEs (%), |FE|s (Hz) and |RFE|s (Hz/s) returned by the e-IpD${}^{2}$FT and IpD${}^{2}$FT in the “frequency deviation + second harmonic + modulation” test. The meaning of the gray cells is the same as in Table 4.

Parm.	Std.	c	Hanning		Hamming	
			e-IpD${}^{2}$FT	IpD${}^{2}$FT	e-IpD${}^{2}$FT	IpD${}^{2}$FT
TVE	3	2	2.25	8.40	0.62	7.99
		3	0.23	2.78	0.19	1.88
|FE|	0.3	2	0.40	1.35	0.14	1.29
		3	0.04	0.15	0.04	0.16
|RFE|	14	2	134.41	540.20	19.30	482.36
		3	4.12	100.35	5.69	61.10

**Table 6:** TVEs (%), |FE|s (Hz) and |RFE|s (Hz/s) returned by the e-IpD${}^{2}$FT and IpD${}^{2}$FT. The Hanning window is adopted in the e-IpD${}^{2}$FT and IpD${}^{2}$FT. The observation window is three cycles long. The TVE, FE and RFE limits in the standard are the same as in Table 4. The meaning of the gray cells is the same as in Table 4.

Test Type	Method		TVE	|FE|	|RFE|
Fre. Dev. + 2nd Harm + 3rd Harm	e-IpD${}^{2}$FT		0.12	0.01	1.32
	IpD${}^{2}$FT		2.43	0.11	89.09
Fre. Dev. + 2nd Harm + 60 dB Noise	e-IpD${}^{2}$FT	mean	0.09	0.00	0.33
		std. dev.	0.00	0.00	0.06
	IpD${}^{2}$FT	mean	2.38	0.06	55.93
		std. dev.	0.00	0.00	712.73

**Table 7:** Results (maximum values) returned by the e-IpD${}^{2}$FT and IpD${}^{2}$FT under different sampling rates. The TVE, FE and RFE limits in the standard are the same as in Table 4.

Parm.	Method	Sampling Rate (Hz)			
		2000	2400	4000	4800
TVE	e-IpD${}^{2}$FT	0.04	0.04	0.04	0.04
	IpD${}^{2}$FT	0.06	0.06	0.07	0.07
|FE|	e-IpD${}^{2}$FT	0.00	0.00	0.00	0.00
	IpD${}^{2}$FT	0.01	0.01	0.01	0.01
|RFE|	e-IpD${}^{2}$FT	0.06	0.06	0.06	0.06
	IpD${}^{2}$FT	2.04	2.13	2.30	2.34

**Table 8:** Harmonic components of the test signal.

Harm. Comp.	2nd	3rd	4th	5th	6th	7th
Magnitude (% of the fundamental)	63	26.8	5.1	4.1	3.7	2.4

**Table 9:** TVEs (%), |FE|s (Hz) and |RFE|s (Hz/s) of the two methods. The Hanning window is adopted in both methods. The observation window is three cycles long. The TVE, FE and RFE Limits in the standard are the same as in Table 4. The meaning of the gray cells is the same as in Table 4.

Test Type	Method		TVE	|FE|	|RFE|
Fre. Dev. + Harmonics + 60 dB Noise	e-IpD${}^{2}$FT	mean	0.55	0.01	0.98
		std. dev.	0.00	0.00	0.36
	IpD${}^{2}$FT	mean	1.86	0.12	41.94
		std. dev.	0.00	0.00	398.88

## Appendix A. Proof of Equations (16)–(18)

A stationary voltage/current signal containing the fundamental and second harmonic components can be defined as

(A1) x(t)=x1(t)+x2(t)=a1cos(2πf1t+ϕ1)+a2cos(2π·2f1·t+ϕ2)=Re{2p1ej2πf1t+2p2ej2π·2f1·t}

where $a_{1}$ and $a_{2}$ denote the amplitudes of the fundamental and the second harmonic, respectively; $\phi_{1}$ and $\phi_{2}$ denote the phases of the fundamental and the second harmonic, respectively; $p_{1}=\frac{a_{1}}{\sqrt{2}}e^{j\phi_{1}}$ and $p_{2}=\frac{a_{2}}{\sqrt{2}}e^{j\phi_{2}}$ are the fundamental and the second harmonic phasors, respectively; and $x_{1}\left(t\right)=a_{1}\cos\left(2\pi f_{1}t+\phi_{1}\right)$ and $x_{2}\left(t\right)=a_{2}\cos\left(2\pi \cdot 2f_{1}\cdot t+\phi_{2}\right)$ denote the fundamental and the second harmonic, respectively. Assume $\Delta f_{1}$ is the static fundamental frequency deviation, i.e., $\Delta f_{1}=f_{1}-f_{0}$ (with $f_{0}$ being the nominal fundamental frequency). According to the IEEE standard [1], the synchrophasor is defined as a phasor referred to the nominal fundamental frequency, i.e., $s_{1}\left(t\right)=p_{1}e^{j2\pi \Delta f_{1}t}$. Please note that due to the possible frequency deviation, the synchrophasor $s_{1}\left(t\right)$ should be a time function, even though the fundamental phasor $p_{1}$ is a constant. Because $\Delta f_{1}$ is a constant, the *i*-th order derivative of the synchrophasor $s_{1}\left(t\right)$ is

(A2) $$s_{1}^{\left(i\right)}\left(t\right)={\left(j2\pi \Delta f_{1}\right)}^{i}p_{1}\left(t\right)\;i=0,1,2$$

Then, the synchrophasor derivative estimates are as follows:

(A3) $$\begin{array}{cc} {\hat{s}}_{1}^{\left(i\right)}\left(t\right) & =x\left(t\right)⊗h_{i}\left(t\right)\\ & =x_{1}\left(t\right)⊗h_{i}^{d}\left(t\right)+x_{1}\left(t\right)⊗\left(h_{i}\left(t\right)-h_{i}^{d}\left(t\right)\right)+x_{2}\left(t\right)⊗h_{i}\left(t\right)\\ & =s_{1}^{\left(i\right)}\left(t\right)+\eta_{i}\left(t\right)\;i=0,1,2 \end{array}$$

where $h_{i}^{d}\left(t\right)$ is the ideal filter for the estimation of the *i*-th order synchrophasor derivative, it should have ideal frequency responses as follows:

(A4) $$H_{i}\left(f\right)=\left\{\begin{array}{c} \sqrt{2}{\left[j2\pi \left(f-f_{0}\right)\right]}^{i}\;\left|f-f_{0}\right|\leq 5\\ 0\;\;\;\;\;|f-f_{0}|>5 \end{array}\right.$$

and $\eta_{i}\left(t\right)$ is the estimation error of $p_{1}^{\left(i\right)}\left(t\right)$, which is given by

(A5) $$\eta_{i}\left(t\right)=x_{1}\left(t\right)⊗\left(h_{i}\left(t\right)-h_{i}^{d}\left(t\right)\right)+x_{2}\left(t\right)⊗h_{i}\left(t\right)\;i=0,1,2$$

Additionally, according to Equations (A3) and (A5), we can obtain

(A6) $${\hat{s}}_{1}^{*}\left(t\right){\hat{s}}_{1}^{\left(i\right)}\left(t\right)=s_{1}^{*}\left(t\right)p_{1}^{\left(i\right)}\left(t\right)+\varepsilon_{i}\left(t\right)\;i=0,1,2$$

where $\varepsilon_{i}\left(t\right)$ is the difference between ${\hat{s}}_{1}^{*}\left(t\right){\hat{s}}_{1}^{\left(i\right)}\left(t\right)$ and $s_{1}^{*}\left(t\right)s_{1}^{\left(i\right)}\left(t\right)$, which is given by

(A7) $$\begin{array}{cc} \varepsilon_{i}\left(t\right) & =s_{1}^{*}\left(t\right)\eta_{i}\left(t\right)+s_{1}^{\left(i\right)}\left(t\right)\eta_{0}^{*}\left(t\right)+\eta_{0}^{*}\left(t\right)\eta_{i}\left(t\right)\\ & =s_{1}^{*}\left(t\right)\eta_{i}\left(t\right)+{\left(j2\pi \Delta f_{1}\right)}^{i}s_{1}\left(t\right)\eta_{0}^{*}\left(t\right)+\eta_{0}^{*}\left(t\right)\eta_{i}\left(t\right)\;i=0,1,2 \end{array}$$

According to Equations (9) and (10), after some algebra, the TVE, FE and RFE of the estimator can be given by [23]

(A8) $$TVE=|\frac{\eta_{0}\left(t\right)}{s_{1}\left(t\right)}|$$

(A9) FE=Δf1Re{η0(t)s1(t)}+12πIm{η1(t)s1(t)}+o(η)

(A10) RFE=2πΔf12Im{η0(t)s1(t)}−2Δf1Im{η1(t)s1(t)}+12πIm{η2(t)s1(t)}+o(η)

where o(η) denotes negligible high-order term of $\eta_{i}$. Thus, we can obtain

(A11) $$\begin{array}{cc} TVE & =\frac{\sqrt{2}}{2}\left|\frac{\left(H_{0}\left(f_{1}\right)-\sqrt{2}\right)p_{1}+H_{0}\left(-f_{1}\right)p_{1}^{*}+H_{0}\left(2f_{1}\right)p_{2}+H_{0}\left(-2f_{1}\right)p_{2}^{*}}{s_{1}\left(t\right)}\right|\\ & \leq \frac{\sqrt{2}}{2}\left\{\right|H_{0}\left(f_{1}\right)-\sqrt{2}|\frac{|p_{1}|}{|s_{1}\left(t\right)|}+|H_{0}\left(-f_{1}\right)|\frac{|p_{1}^{*}|}{|s_{1}\left(t\right)|}+|H_{0}\left(2f_{1}\right)|\frac{|p_{2}|}{|s_{1}\left(t\right)|}+|H_{0}\left(-2f_{1}\right)\left|\frac{|p_{2}^{*}|}{|s_{1}\left(t\right)|}\right\}\\ & \leq \frac{\sqrt{2}}{2}\left\{\right|H_{0}\left(f_{1}\right)-\sqrt{2}\left|+\right|H_{0}\left(-f_{1}\right)\left|+r[\right|H_{0}\left(2f_{1}\right)\left|+\right|H_{0}\left(-2f_{1}\right)\left|]\right\} \end{array}$$

(A12) FE=22Δf1·Re{(H0(f1)−2)p1+H0(−f1)p1*+H0(2f1)p2+H0(−2f1)p2*s1(t)}+22·12πIm{[H1(f1)−2(j2πΔf1)]p1+H1(−f1)p1*+H1(2f1)p2+H1(−2f1)p2*s1(t)}≤22|Δf1|·|(H0(f1)−2)p1+H0(−f1)p1*+H0(2f1)p2+H0(−2f1)p2*s1(t)|+22·12π|[H1(f1)−2(j2πΔf1)]p1+H1(−f1)p1*+H1(2f1)p2+H1(−2f1)p2*s1(t)|≤22|Δf1|{|(H0(f1)−2)|+|H0(−f1)|+r(|H0(2f1)|+|H0(−2f1)|)}+22·12π{|[H1(f1)−2(j2πΔf1)]|+|H1(−f1)|+r(|H1(2f1)|+|H1(−2f1)|)}

(A13) RFE=22·2πΔf12·Im{(H0(f1)−2)p1+H0(−f1)p1*+H0(2f1)p2+H0(−2f1)p2*s1(t)}−22·2Δf1Im{[H1(f1)−2(j2πΔf1)]p1+H1(−f1)p1*+H1(2f1)p2+H1(−2f1)p2*s1(t)}+22·12πIm{(H2(f1)+2·4π2Δf12)p1+H2(−f1)p1*+H2(2f1)p2+H2(−2f1)p2*s1(t)}≤22·2πΔf12{|(H0(f1)−2)|+|H0(−f1)|+r(|H0(2f1)|+|H0(−2f1)|)}+22|2Δf1|{|[H1(f1)−2(j2πΔf1)]|+|H1(−f1)|+r(|H1(2f1)|+|H1(−2f1)|)}+22·12π{|(H2(f1)+2·4π2Δf12)|+|H2(−f1)|+r(|H2(2f1)|+|H2(−2f1)|)}

where $H_{i}\left(f_{1}\right)$ (with i=0,1,2) represents the frequency response of $h_{i}\left[n\right]$ (with i=0,1,2) at the fundamental frequency $f_{1}$; and $r=\frac{|p_{2}|}{|p_{1}|}=\frac{|p_{2}|}{|s_{1}\left(t\right)|}$ is the magnitude ratio of the second harmonic to the fundamental. Please note that the denominator in Equations (A11)–(A13) is reduced since $|p_{1}\left|=\right|p_{1}^{*}\left|=\right|s_{1}\left(t\right)|$. Additionally, the “Im{·}” part becomes “|·|” because the imaginary part of a phasor is always not greater than its absolute value.

When $f_{1}=f_{0}$, $\Delta f_{1}=0$. Then, Equations (A11)–(A13) can be rewritten or simplified as

(A14) $$TVE\leq \frac{\sqrt{2}}{2}\left\{\right|H_{0}\left(f_{0}\right)-\sqrt{2}\left|+\right|H_{0}\left(-f_{0}\right)\left|+r(\right|H_{0}\left(2f_{0}\right)\left|+\right|H_{0}\left(-2f_{0}\right)\left|)\right\}$$

(A15) $$FE\leq \frac{\sqrt{2}}{2}\cdot \frac{1}{2\pi }\left\{\right|H_{1}\left(f_{0}\right)\left|+\right|H_{1}\left(-f_{0}\right)\left|+r(\right|H_{1}\left(2f_{0}\right)\left|+\right|H_{1}\left(-2f_{0}\right)\left|)\right\}$$

(A16) $$RFE\leq \frac{\sqrt{2}}{2}\cdot \frac{1}{2\pi }\left\{\right|H_{2}\left(f_{0}\right)\left|+\right|H_{2}\left(-f_{0}\right)\left|+r(\right|H_{2}\left(2f_{0}\right)\left|+\right|H_{2}\left(-2f_{0}\right)\left|)\right\}$$

## Appendix B. Proof of Equations (19)–(21)

For a filter given in Equation (15)

(A17) $$\begin{array}{c} h_{i}\left[n\right]=\sum_{q=1}^{3}R_{i}\left[q\right]\cdot g_{q}\left[n\right]+\sum_{q=4}^{6}R_{i}\left[q\right]\cdot g_{q-3}^{*}\left[n\right]\;i=0,1,2 \end{array}$$

its frequency response at frequency $f_{0}$ is

(A18) $$\begin{array}{cc} H_{i}\left(f_{0}\right) & =\sum_{n=-(N_{w}-1)/2}^{(N_{w}-1)/2}h_{i}\left[n\right]e^{-j2\pi f_{0}n/f_{s}}\\ & =\sum_{n=-(N_{w}-1)/2}^{(N_{w}-1)/2}\left(\sum_{q=1}^{3}R_{i}\left[q\right]\cdot g_{q}\left[n\right]\right)e^{-j2\pi f_{0}n/f_{s}}\\ & +\sum_{n=-(N_{w}-1)/2}^{(N_{w}-1)/2}\left(\sum_{q=4}^{6}R_{i}\left[q\right]\cdot g_{q-3}^{*}\left[n\right]\right)e^{-j2\pi f_{0}n/f_{s}}\\ & =\sum_{q=1}^{3}R_{i}\left[q\right]\sum_{n=-(N_{w}-1)/2}^{(N_{w}-1)/2}g_{q}\left[n\right]e^{-j2\pi f_{0}n/f_{s}}\\ & +\sum_{q=4}^{6}R_{i}\left[q\right]\sum_{n=-(N_{w}-1)/2}^{(N_{w}-1)/2}g_{q-3}^{*}\left[n\right]e^{-j2\pi f_{0}n/f_{s}}\\ & =\sqrt{2}\sum_{q=1}^{3}R_{i}\left[q\right]W_{0}\left(-\left(f_{q}-f_{0}\right)\right)+\sqrt{2}\sum_{q=4}^{6}R_{i}\left[q\right]W_{0}\left(f_{q-3}+f_{0}\right)\\ & =\sqrt{2}\sum_{q=1}^{3}R_{i}\left[q\right]W_{0}\left(f_{q}-f_{0}\right)+\sqrt{2}\sum_{q=4}^{6}R_{i}\left[q\right]W_{0}\left(f_{q-3}+f_{0}\right)\;i=0,1,2 \end{array}$$

where $W_{0}\left(-\left(f_{q}-f_{0}\right)\right)=W_{0}\left(f_{q}-f_{0}\right)$ because $W_{0}\left(f\right)$ is an even function. Please note that $R_{i}\left[q\right]$ is an element of $W^{-1}$, where

(A19) $$\begin{array}{c} W^{-1}=\left[\begin{array}{cccc} R_{0}\left[1\right] & R_{0}\left[2\right] & \cdots & R_{0}\left[6\right]\\ R_{1}\left[1\right] & R_{1}\left[2\right] & \cdots & R_{1}\left[6\right]\\ \vdots & \vdots & \ddots & \vdots \\ R_{5}\left[1\right] & R_{5}\left[2\right] & \cdots & R_{5}\left[6\right] \end{array}\right] \end{array}$$

Because W has the form of

(A20) $$\begin{array}{c} W=\left[\begin{array}{cccccc} W_{0}\left(f_{1}-f_{0}\right) & W_{1}\left(f_{1}-f_{0}\right) & W_{2}\left(f_{1}-f_{0}\right) & W_{0}\left(f_{1}+f_{0}\right) & W_{1}\left(f_{1}+f_{0}\right) & W_{2}\left(f_{1}+f_{0}\right)\\ W_{0}\left(f_{2}-f_{0}\right) & W_{1}\left(f_{2}-f_{0}\right) & W_{2}\left(f_{2}-f_{0}\right) & W_{0}\left(f_{2}+f_{0}\right) & W_{1}\left(f_{2}+f_{0}\right) & W_{2}\left(f_{2}+f_{0}\right)\\ W_{0}\left(f_{3}-f_{0}\right) & W_{1}\left(f_{3}-f_{0}\right) & W_{2}\left(f_{3}-f_{0}\right) & W_{0}\left(f_{3}+f_{0}\right) & W_{1}\left(f_{3}+f_{0}\right) & W_{2}\left(f_{3}+f_{0}\right)\\ W_{0}^{*}\left(f_{1}+f_{0}\right) & W_{1}^{*}\left(f_{1}+f_{0}\right) & W_{2}^{*}\left(f_{1}+f_{0}\right) & W_{0}^{*}\left(f_{1}-f_{0}\right) & W_{1}^{*}\left(f_{1}-f_{0}\right) & W_{2}^{*}\left(f_{1}-f_{0}\right)\\ W_{0}^{*}\left(f_{2}+f_{0}\right) & W_{1}^{*}\left(f_{2}+f_{0}\right) & W_{2}^{*}\left(f_{2}+f_{0}\right) & W_{0}^{*}\left(f_{2}-f_{0}\right) & W_{1}^{*}\left(f_{2}-f_{0}\right) & W_{2}^{*}\left(f_{2}-f_{0}\right)\\ W_{0}^{*}\left(f_{3}+f_{0}\right) & W_{1}^{*}\left(f_{3}+f_{0}\right) & W_{2}^{*}\left(f_{3}+f_{0}\right) & W_{0}^{*}\left(f_{3}-f_{0}\right) & W_{1}^{*}\left(f_{3}-f_{0}\right) & W_{2}^{*}\left(f_{3}-f_{0}\right) \end{array}\right] \end{array}$$

Then, we can obtain

(A21) $$\begin{array}{c} H_{0}\left(f_{0}\right)=\sqrt{2}\left\{\sum_{q=1}^{3}R_{0}\left[q\right]W_{0}\left(f_{q}-f_{0}\right)+\sum_{q=4}^{6}R_{0}\left[q\right]W_{0}\left(f_{q-3}+f_{0}\right)\right\}=\sqrt{2} \end{array}$$

(A22) $$\begin{array}{c} H_{1}\left(f_{0}\right)=\sum_{q=1}^{3}R_{1}\left[q\right]W_{0}\left(f_{q}-f_{0}\right)+\sum_{q=4}^{6}R_{1}\left[q\right]W_{0}\left(f_{q-3}+f_{0}\right)=0 \end{array}$$

(A23) $$\begin{array}{c} H_{2}\left(f_{0}\right)=\sum_{q=1}^{3}R_{2}\left[q\right]W_{0}\left(f_{q}-f_{0}\right)+\sum_{q=4}^{6}R_{2}\left[q\right]W_{0}\left(f_{q-3}+f_{0}\right)=0 \end{array}$$

The above three equations correspond to the first three elements of the first column of the identity matrix $I=W^{-1}W$. According to Equation (A21), we have

(A24) $$\begin{array}{c} |H_{0}\left(f_{0}\right)-\sqrt{2}|=0 \end{array}$$

Additionally, when $|f-f_{0}|>f_{0}$, the attenuation of the estimator is very high. Thus, we can obtain

(A25) $$\left\{\begin{array}{c} |H_{i}\left(-f_{0}\right)|\approx 0\\ |H_{i}\left(-2f_{0}\right)|\approx 0\;i=0,1,2 \end{array}\right.$$

According to Equations (A22)–(A25), Equations (16)–(18) can be simplified as

(A26) $$\begin{array}{c} TVE\leq \frac{\sqrt{2}}{2}\cdot r\left|H_{0}\left(2f_{0}\right)\right| \end{array}$$

(A27) $$\begin{array}{c} FE\leq \frac{\sqrt{2}}{2}\cdot \frac{r}{2\pi }\left|H_{1}\left(2f_{0}\right)\right| \end{array}$$

(A28) $$\begin{array}{cc} RFE & \leq \frac{\sqrt{2}}{2}\cdot \frac{r}{2\pi }\left|H_{2}\left(2f_{0}\right)\right| \end{array}$$
